# Assessment of erythrocyte acetylcholine esterase activities in painters

**DOI:** 10.4103/0019-5278.50720

**Published:** 2009-04

**Authors:** Mohd Imran Khan, Abbas Ali Mahdi, Najmul Islam, Subodh Kumar Rastogi, M. P. S. Negi

**Affiliations:** 1 Department of Biochemistry, C. S. M. Medical University, Lucknow, India; 2 Department of Biochemistry, J. N. Medical University, Aligarh Muslim University, Aligarh, India; 3 Emeritus Scientist, Indian Institute of Toxicology and Research, Lucknow, India; 4 Biometry Division, Central Drug Research Institute, Lucknow, India

**Keywords:** AChE in red blood cell and plasma, blood lead, painters

## Abstract

Thirty-five male painters in the age group of 20–50 years occupationally engaged in domestic and commercial painting for 5–12 years having blood lead levels (BLL) ≤40 μg/dl were subjected to the determination of acetyl choline esterase (AChE) levels both in plasma and red blood cell (RBC) lysate. BLL were determined using a graphite furnace atomic absorption spectrometer. The results showed that BLL were 7.7 times higher in the painters as compared with that of the control group. Significant decreases in RBC and plasma AChE were observed in the exposed group in comparison with controls. RBC and plasma AChE showed a decrease of 18.4% and 18%, respectively, in the exposed group. The findings also indicated a significant negative correlation of both RBC and plasma AChE activities with BLL. The marked reduction observed in both RBC and plasma AChE activity may account for disruption of cholinergic function and result in neurotoxicity among the painters.

## INTRODUCTION

Lead, a metal used by mankind for over 6,000 years, is one of the most widely scattered toxic metals in the environment today. The metal does not have any known useful function in the human body and produces harmful effects once it enters inside the body either by ingestion, inhalation, or by dermal contact (organic lead). Although the occupational safety and health administration (O.S.H.A.) permissible limit for occupational lead exposure is < 40μg/dl, adverse health effects below this standard have been reported. Neurobehavioral, hematologic, nephrotoxic, reproductive, and cardiovascular effects of lead have been observed in humans and in other animals.[1–7]

Numerous studies in humans and animals indicate that lead exposure can promote brain dysfunction, which may lead to behavioral changes and altered cognitive function. Lead preferentially affects the central nervous system; moreover, it also poisons the motor neurons within the peripheral nervous system.[8–11] Although the neurotoxicity of lead has been extensively documented by numerous studies, most of these studies have relied on neurobehavioral or neurophysiologic methods in assessing the neurotoxicity of lead.[12–14] Recent evidences suggest that the neurotoxic events during lead toxicity may be mediated through an interference with the cholinergic system, suggesting that this altered biochemical intracellular communication may lead toward the well-known neurobehavioral and neurophysical end point usually observed in lead neurotoxicity.[15,16] The cholinergic system plays a pivotal role in many of the central nervous system functions, including cognitive behavior. Impairment of this system appears to be associated with the behavioral disturbances and deficit in learning and memory usually observed in humans and animals.[17,18] Cholinergic neurotransmission impairment of the brain has already been well stabilized in experimental animals. However, a similar study of occupational or environmental settings has not been well understood in humans mainly because of ethical considerations. The recent accumulating experimental evidences however point that biochemical signals of the neurotransmission system similar to those involved as neurotoxic targets in the central nervous system are also present in more easily and ethically obtainable body fluids like peripheral blood cells, urine, saliva, and cerebrospinal fluid and can thus be used in assessing exposure and response of the central nervous system when functional damage may not yet be apparent.[19] In this context, plasma and red blood cell (RBC) acetylcholine esterase (AChE) are currently being extensively investigated in human as surrogate biomarkers of exposure and for predicting the neurotoxic effects of toxicants.[17,18,20]

Lead is widely used in various paints because of its anticorrosive properties and ability to hold pigments together. Recently, Alphen *et al*. reported that 10% of lead metal consumed in India is used for manufacturing of paints.[21] Another study by Clark et al. had reported that 100% of Indian paints are known to contain lead at levels above the US limit of 0.06%.[22] Painters are continuously exposed to these lead-containing paints as well as to an extensive variety of hazardous substances like organic solvents and residual plastic monomers. In the present study, we have assessed the blood lead levels (BLL) of painters using commercially available Indian paints and occasionally gasoline or thinners as solvents. The application of paints was performed with brushes or by aerosol spraying while the removal was performed by sandblasting and scrapping of the surfaces previously coated with paint. Both the tasks were performed without proper protection (e.g., wearing gloves and masks).

## MATERIALS AND METHODS

### Selection of the study population

For the study, the ethical clearance was obtained from King George's Medical University, Lucknow, India. A written informed consent was taken from subjects by explaining the importance of the study in their local language. Residential and commercial painters were selected and recruited on the basis of routine surveillance data of those who were engaged with local contractors in Lucknow city. A population of 53 male painters in the age group of 20–50 years was screened for BLL, of which 35 painters who had BLL ≤40 μg/dl were selected for this study. These painters were engaged in house painting for 8–9 h a day and were performing this for a period of 5–10 years. An equal number of controls of the similar age group belonging to the similar socioeconomic status with no occupational exposure to lead and who were normal healthy are selected from the same vicinity. Information on occupation and medical history, job description, socioeconomic status, and lifestyle of both groups were obtained through questionnaire. Subjects having a previous history of metabolic diseases such as diabetes mellitus, hypertension, malignancy, heart disease, etc, infectious diseases like arthritis and tuberculosis, and endocrinal disorders as well as other conditions known to generate oxidative stress were excluded from the study. Ten painters who had initially joined the study discontinued on their own while eight painters on clinical examination were found to suffer from metabolic diseases such as diabetes and heart diseases.

### Blood collection

By taking all aseptic precautions, 5 ml of total venous blood is collected in dipotassium EDTA vacationers (BD Biosciences, San Jose, CA, USA) from each subject and control by an expert phlebotomist. Two milliliters of whole venous blood is used for the estimation of lead. The other 3 ml of blood is centrifuged at 3000 rpm for 15 min for separation of plasma. The remaining packed RBCs were lysed by mixing normal saline and both plasma and RBCs lysate were used for the estimation of AChE.

### Analytical methods

BLL were determined using a graphite furnace atomic absorption spectrometer (Varian Australia Pty, Victoria, Australia).[23] The instrument was calibrated using aqueous standards of various lead concentrations (10–40 *μ*g). The AChE levels were determined in plasma and RBC lysate according to the method of Venkatramana *et al*.[24] AChE (true and pseudo) estimation was performed by the colorimetric method using acetylcholine chloride as the substrate.

### Statistical analysis

Student's *t*-test was used to analyze the significance of the mean differences between control subjects and lead-exposed painters. Relative association among parameters of control subjects and lead-exposed painters were determined separately by Karl Pearson's correlation coefficient (r). Linear regression analysis was performed to determine the strength of the relationship of BLL with other significantly correlated parameters of lead-exposed painters. The value *P* <0.05 was considered significant and *P* <0.01 was considered very significant. However, *P* >0.05 was considered as not significant.

## RESULTS

Table 1 summarizes the demographic data of both painters and control subjects. The average professional years as a painter were about 9 years (range 5–12 years). The working conditions for these painters remained fairly stable without significant changes over the past several years. The working years in the control group averaged 7 years (range 5–10 years). The parameters of smoking and alcohol consumptions did not differ significantly between the two groups.

**Table 1:** Demographic data of subjects

Variables	Painters (*n* = 35)	Controls (*n* = 35)
Age (years)	38.1 ± 1.54	36.8 ± 1.46
Years in employments	5–12	5–10
Initial age at employment (years)	22.0 ± 3.23	21.3 ± 3.02
Smoking (pack–years)	81.8 ± 106.3 (54.0%)	72.2 ± 90.01 (52.0%)
Alcohol (ml–days)	35.1 ± 52.53 (39.1%)	39.4 ± 56.90 (40.0%)

Data represent mean ± S.D, where *P <0.01 was considered significant.

Table 2 shows BLL and AChE activities in both RBC and plasma. BLL were found to be 7.3-fold higher in painters when compared with controls. Significant (*P* < 0.01) decreases in RBC and plasma AChE were observed in painters in comparison with controls. RBC and plasma AChE showed a decrease of 18.4% and 18%, respectively, from the control group.

**Table 2:** Summary of parameters of controls and painters

Parameters	Control (n = 35)	Painters (n = 35)
Blood Pb (*μ*g/dl)	2.79 ± 0.16	21.64 ± 1.02*
RBC AChE (U/ml)	308.30 ± 1.24	252.00 ± 3.16*
Plasma AChE (U/ml)	153.47 ± 2.22	125.77 ± 1.38*

* *P* < 0.01 was considered significant.

Table 3 depicts significant (*P* < 0.01) negative correlation of both RBC and plasma AChE with BLL of painters. However, RBC AChE shows higher negative correlation (r = -0.88, *P* < 0.01) than plasma (r= -0.75, *P* < 0.01). Similar negative correlation (r= -0.71, *P* <0.01) was observed between RBC and plasma AChE.

**Table 3:** Pearson's correlation (r) between blood lead and AChE (RBC and plasma)

Parameters	Blood Pb	RBC AChE	Plasma AChE
Blood Pb	1.00		
RBC AChE	-0.88**	1.00	
Plasma AChE	-0.75**	-0.71**	1.00

** *P* < 0.01 was considered significant.

**Figure 1:**
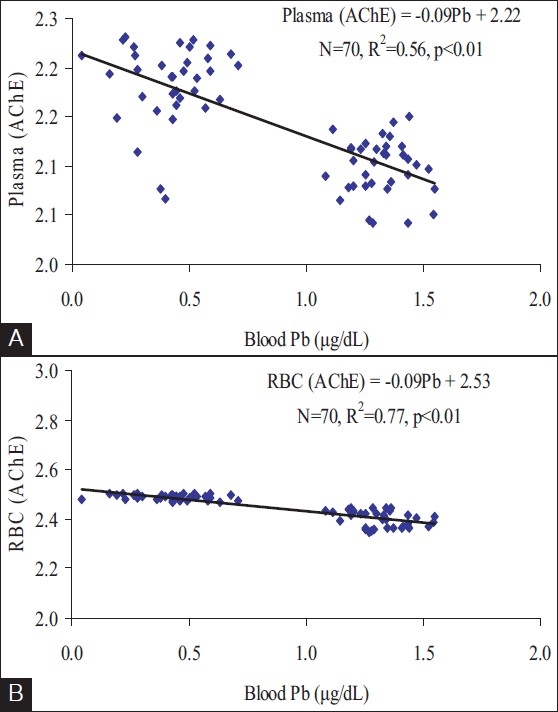
(A) Best-fit regression between blood Pb and plasma AChE (B) Best-fit regression between blood Pb and RBC AChE

Figure 1 shows best-fit regression graphs of RBC and plasma AChE activities with BLL. RBC AChE shows higher affinity with BLL of painters than plasma AChE.

## DISCUSSION

AChE is an enzyme that is responsible for interrupting normal nerve transmission at the synapse by hydrolyzing the neurotransmitter Ach to acetic acid and choline. This transmission interruption ensures that the nervous system is not unnecessarily stimulated. In our study, we observed that AChE activity both in RBCs and plasma was decreased significantly in painters (18.4% and 18%, respectively) as compared with the control unexposed group. Besides this, a negative correlation of both RBCs and plasma AChE with BLL was also recorded in the painters. Painters are excessively exposed to lead while scrapping the old paint and painting the scrubbed surfaces. Lead is widely used in various paints because of its anticorrosive properties and ability to hold pigments together. Alphen *et al*. reported that nearly 10% of lead consumed in India is used for manufacturing of paints.[21] Our findings confirm the earlier findings that lead exposure mainly affects the cholinergic system by reducing Ach release as lead competes with calcium (Ca^2+^), which may account for the disruption of the cholinergic function and alteration in the other transmitter systems.[25–27] Several pharmacological studies have also demonstrated marked reduction in cholinergic function.[28–31] The nervous system is the primary target for the Pb exposure and it is therefore vulnerable to Pb neurotoxicity.[32,33] The mechanism by which lead inhibits AChE is still not well known. One of the best characterized toxic effects of lead is its inhibition of the enzyme delta-aminolevulinic acid dehydratase (ALAD), which is effected by lead binding to the free sulphydryl groups on ALAD-active sites.[34] Inhibition of ALAD results in the elevation of delta-aminolevulinic acid (ALA) in the blood.[35,36] ALA undergoes autooxidation at physiological pH, resulting in the formation of superoxide, hydrogen peroxide, and ALA free radicals. Recent studies by Tsakiris *et al*. indicate that the activity of AChE is decreased on exposure to free radicals.[37] It is tempting to speculate that the inhibitory effect of lead on AChE observed in the painters in this study might be due to free radicals produced by lead. Lead also has a high affinity for free -SH groups present in enzymes and proteins and its binding can alter their function.[38,39] This may also lead to the observed inhibition of AChE activity and consequent increase in Ach level in the present study, which is in agreement with the earlier reports.[40,41] Accumulation of ACh at synaptic regions could lead to desensitization of the cholinergic receptors on the post-synaptic membrane, rendering it ineffective to the transmitter action.[27]

## CONCLUSION

The study conducted on painters revealed that Pb exposure remarkably decreases the RBCs and plasma AChE activity. Both RBC and plasma AChE showed negative correlation with BLL. The decrease in both RBC and plasma AChE is indicative of disruption of the cholinergic function, leading to neurotoxicity in exposed workers.

## References

[1] Ademuyiwa O, Ugbaja RN, Ojo DA, Owoigbe AO, Adeokun SE (2005). Reversal of aminolevulinic acid dehydratase (ALAD) inhibition and reduction of erythrocyte protoporphyrin levels by vitamin C in occupational lead exposure in Abeokuta, Nigeria. Environ Toxicol Pharmacol.

[2] Canfield RL, Henderson CR, Cory-Slechta DA, Cox C, Jusko TA, Lanphear BP (2003). Intellectual impairment in children with blood lead concentrations below 10μg per deciliter. N Engl J Med.

[3] Cory-Slechta DA (2003). Lead-induced impairments in complex cognitive function: Offerings from experimental studies. Child Neuropsychol.

[4] Diamond GL, Tarloff J, Lash L (2005). Risk assessment of nephrotoxic metals. The Toxicology of the Kidney.

[5] Glenn BS, Stewart WF, Links JM, Todd AC, Schwartz BS (2003). The longitudinal association of lead with blood pressure. Epidemiology.

[6] Sallmén M, Lindbohm ML, Nurminen M (2000). Paternal exposure to lead and infertility. Epidemiology.

[7] Vaziri ND, Sica DA (2004). Lead-induced hypertension: role of oxidative stress. Curr Hypertens Rep.

[8] Banks EC, Ferretti LE, Shucard DW (1997). Effect of low level lead exposure on cognitive function in children: A review of behavioral, neurophysiological and biological evidence. Neurotoxicology.

[9] Needleman HL, Schell A, Bellinger D, Leviton A, Allred EN (1990). The long-term effects of exposure to lead in childhood: An 11-year follow-up report. N Engl J Med.

[10] Nehru B, Sidhu P (2001). Behavior and neurotoxic consequences of lead on rat brain followed by recovery. Biol Trace Elem Res.

[11] Seppäläinen AM, Hernberg S, Vesanto R, Kock B (1983). Early neurotoxic effects of occupational lead exposure: A prospective study. Neurotoxicology.

[12] Anger WK (1990). Worksite behavioral research: Results, sensitive methods, test batteries and the transition from laboratory data to human health. Neurotoxicology.

[13] Costa LG, Manzo L (1995). Biochemical markers of neurotoxicity: research strategies and epidemiological applications. Toxicol Lett.

[14] Hänninen H, Aitio A, Kovala T, Luukkonen R, Matikainen E, Mannelin T (1998). Occupational exposure to lead and neuropsychological dysfunction. Occup Environ Med.

[15] Bressler JP, Goldstein GW (1991). Mechanism of lead neurotoxicity. Biochem Pharmacol.

[16] De Roos FJ, Greenberg MI, Hamilton RJ, Phillips SD, McCluskey GJ (2003). Smelters and metal reclaimers. Occupational, Industrial, and Environmental Toxicology.

[17] Castoldi AF, Coccini T, Rossi A, Nicotera P, Costa LG, Tan XX (1994). Biomarkers in environmental medicine: Alterations of cell signaling as early indicators of neurotoxicity. Funct Neurol.

[18] Manzo L, Castoldi AF, Coccini T, Rossi AD, Nicotera P, Costa LG (1995). Mechanisms of neurotoxicity: Applications to human biomonitoring. Toxicol Lett.

[19] Manzo L, Artigas F, Martínez E, Mutti A, Bergamaschi E, Nicotera P (1996). Biochemical markers of neurotoxicity: A review of mechanistic studies and applications. Hum Exp Toxicol.

[20] Ademuyiwa O, Ugbaja RN, Idumebor F, Adebawo O (2005). Plasma lipid profiles and risk of cardiovascular disease in occupational lead exposure in Abeokuta, Nigeria. Lipids Health Dis.

[21] Alphen MV (1999). Lead in paints and water in India.

[22] Clark CS, Rampal KG, Thuppil V, Chen CK, Clark R, Roda S (2006). The lead content of currently available new residential paint in several Asian countries. Environ Res.

[23] Aguilera de Benzo Z, Fraile R, Carrison N (1989). Determination of lead in whole blood by electrothermal atomization atomic absorbance spectrometry using tube and platform atomizers and dilution with Triton X-100. J Anal Atom spectrom.

[24] Venkataraman BV, Rani MA, Andrade C, Joseph T (1993). Improved colorimetric method for cholinesterase activity. Indian J Physiol Pharmacol.

[25] Cooper GP, Suszkiw JB, Manalis RS, Narahashi T (1984). Presynaptic effects of heavy metals. Cellular and molecular neurotoxicology.

[26] Suszkiw J, Toth G, Murawsky M, Cooper G (1984). Effects of Pb2+ and Cd2+ on acetylcholine release and Ca^2+^ movements in synaptosomes sub cellular fractions from rat brain and torpedo electric organ. Brain Res.

[27] Silbergeld EK (1977). Interaction of lead and calcium on the synaptosomal uptake of dopamine and choline. Life Sci.

[28] Alfano DP, Petit TL (1982). Neonatal lead exposure alters dendritic development of hippocampal granule cells. Exp Neurol.

[29] Bornschein RL, Fox DA, Michaelson IA (1977). Estimation of daily exposure in neonatal rats receiving lead via dam's milk. Toxicol Appl Pharamcol.

[30] Silbergeld EK, Goldberg AM (1975). Pharmacological and neurochemical investigations of lead induced hyperactivity. Neuropharmacology.

[31] Reddy GR, Basha MR, Devi CB, Suresh A, Baker JL, Shafeek A (2003). Lead induced effects on acetylcholinesterase activity in cerebellum and hippocampus of developing rat. Int J Dev Neurosci.

[32] Basha MR, Wei W, Brydie M, Razmiafshari M, Zawia NH (2003). Lead induced developmental perturbations in hippocampal Sp1 DNA-binding are prevented by zinc supplementation. Int J Dev Neurosci.

[33] Reddy GR, Zawia NH (2000). Lead exposure alters Egr-1 DNA binding in the neonatal rat brain. Int J Dev Neurosci.

[34] Dent AJ, Beyersmann D, Block C, Hasnain SS (1990). Two different zinc sites in bovine 5-amino-levulinate dehydratase distinguished by extended X-ray absorption fine structure. Biochemistry.

[35] Onunkwor B, Dosumu O, Odukoya OO, Arowolo T, Ademuyiwa O (2004). Biomarkers of lead exposure in petrol station attendants and auto-mechanics in Abeokuta, Nigeria: Effects of 2-week ascorbic acid supplementation. Environ Toxicol Pharmacol.

[36] Sakai T (2000). Biomarkers of lead exposure. Ind Health.

[37] Tsakiris S, Angelogianni P, Schulpis KH, Stavridis JC (2000). Protective effect of L-phenylalanine on rat brain acetylcholinesterase inhibition induced by free radicals. Clin Biochem.

[38] Bagchi D, Vuchetich PJ, Bagchi M, Hassoun EA, Tran MX, Tang L (1997). Induction of oxidative stress by chronic administration of sodium dichromate (chromium VI) and cadmium chloride (cadmium II) to rats. Free Rad Biol Med.

[39] Chetty CS, Rajanna S, Hall E, Yallapragada PR, Rajanna B (1996). In vitro and *in vivo* effects of lead, methylmercury and mercury on inositol 1,4,5-triphosphate and 1,3,4 5- tetrakisphosphate receptor bindings in rat brain. Toxicol Lett.

[40] Shih TM, Hanin I (1978). Effect of chronic lead exposure levels of acetylcholine and choline and acetylcholine turnover rate in rat brain areas *in vivo*. Psychopharmacology (Berl).

[41] Gietzen DW, Woolley DE (1984). Acetylcholinesterase activity in the brain of rat pups and dams after exposure to lead via maternal water supply. Neurotoxicology.

